# FGFR Pathway Inhibition in Gastric Cancer: The Golden Era of an Old Target?

**DOI:** 10.3390/life12010081

**Published:** 2022-01-07

**Authors:** Csongor G. Lengyel, Sadaqat Hussain, Andreas Seeber, Sara Jamil Nidhamalddin, Dario Trapani, Baker S. Habeeb, Essam Elfaham, Syed Ayub Mazher, Fahmi Seid, Shah Z. Khan, Khalid El Bairi, Andrew Odhiambo, Sara C. Altuna, Angelica Petrillo

**Affiliations:** 1 Head and Neck Surgery, National Institute of Oncology, 1122 Budapest, Hungary; lengyel.csongor@gmail.com; 2 Oncology Department, University Hospital of Leicester, Leicester LE1 5WW, UK; sadaqat.hussain@uhl-tr.nhs.uk; 3 Comprehensive Cancer Center Innsbruck, Department of Hematology and Oncology, Medical University of Innsbruck, 6020 Innsbruck, Austria; Andreas.seeber@tirol-kliniken.at; 4 Department of Medical Oncology, Hiwa Cancer Hospital, Sulayamaniyah 46001, Iraq; sara.jamil@aol.com; 5 European Institute of Oncology, 20141 Milan, Italy; dario.trapani@ieo.it; 6 Medical Oncology, Shaqlawa Teaching Hospital, Erbil 44001, Iraq; bakershalal@gmail.com; 7 Department of Hematoncology, Kuwait Cancer Control Center (KCCC), Kuwait City 20001, Kuwait; emf86@hotmail.com; 8 Division of Internal Medicine, UT Southwestern Clements University Hospital, Dallas, TX 75390, USA; syed.mazher@utsouthwestern.edu; 9 Department of Oncology, College of Medicine and Health Sciences, Hawassa University, Hawassa 1560, Ethiopia; fahmizeek@gmail.com; 10 Department of Clinical Oncology, BINOR Cancer Hospital, Bannu 28000, Pakistan; skhanizhere0@gmail.com; 11 Cancer Biomarkers Working Group, Oujda 60000, Morocco; k.elbairi@ump.ac.ma; 12 Unit of Medical Oncology, Department of Clinical Medicine, University of Nairobi, Nairobi 00202, Kenya; andrew@uonbi.ac.ke; 13 Oncomédica C. A., Caracas 1040, Venezuela; altunamujica.md@gmail.com; 14 Medical Oncology Unit, Ospedale del Mare, 80147 Naples, Italy

**Keywords:** fibroblast growth factor receptors, FGFR fusions, next generation sequencing, first line, target therapy, FGFR inhibitors, Tyrosine kinase inhibitors, precision medicine, bemarituzumab

## Abstract

Gastric cancer (GC) is the third leading cause of cancer-associated death worldwide. The majority of patients are diagnosed at an advanced/metastatic stage of disease due to a lack of specific symptoms and lack of screening programs, especially in Western countries. Thus, despite the improvement in GC therapeutic opportunities, the survival is disappointing, and the definition of the optimal treatment is still an unmet need. Novel diagnostic techniques were developed in clinical trials in order to characterize the genetic profile of GCs and new potential molecular pathways, such as the Fibroblast Growth Factor Receptor (FGFR) pathway, were identified in order to improve patient’s survival by using target therapies. The aim of this review is to summarize the role and the impact of FGFR signaling in GC and to provide an overview regarding the potential effectiveness of anti-FGFR agents in GC treatment in the context of precision medicine.

## 1. Introduction

Gastric cancer (GC) is the third leading cause of cancer-associated death worldwide following lung and colorectal cancer [1]. Interestingly, its incidence varies geographically across the globe. While in Western countries, the incidence of GC decreased over the past decades, the numbers of newly diagnosed GC cases increased, especially in Asia and Africa [2,3,4]. Next to *Helicobacter Pylori* infection, lifestyle factors, such as alcohol intake and smoking, as well as genetic risk factors have been associated with GC development [5,6,7,8].

Unfortunately, the majority of patients are diagnosed within an advanced stage of disease [9]. In metastatic GC (mGC), systemic chemotherapy remains the standard of care [10], with median overall survival (OS) around 12 months when treated with combinational cytotoxic agents [10]. Thus, to improve patients’ survival, understanding the molecular mechanisms leading to GC development is of great importance.

Histopathological and molecular intra- and intertumoral heterogeneity are major hallmarks of GC and histological classifications, such as Laurén, are not sufficient to stratify patients towards a personalized treatment management [11]. Using novel diagnostic techniques, such as next generation sequencing, the characterization of genetic profile of GCs yielded potential novel therapeutic targets [12,13,14].

One of the first targeted treatments approved in GC was the monoclonal antibody trastuzumab that targets the Human Epidermal Receptor 2 (HER2). HER2 is overexpressed or amplified in 12–20% of all GC cases [15]. Trastuzumab in combination with chemotherapy showed an improved OS and progression-free survival (PFS) in patients harboring a HER2-positive mGC within the phase III ToGA trial [16]. Furthermore, GCs showing a microsatellite instable-high (MSI-H) or a damaged mismatch repair genes (dMMR) status are sensitive to immune checkpoint inhibitor treatment [17]. The anti-Programmed Cell Death 1 (PD-1) antibody pembrolizumab yielded an impressive response and extension of survival in MSI-H/dMMR mGC patients [18].

In this perspective of precision oncology, various new potential molecular pathways could represent novel targets for drug development in GC, such as the Fibroblast Growth Factor Receptor (FGFR) one [19]. Therefore, this review aims to summarize the role and impact of FGFR signaling and to highlight the effectiveness of anti-FGFR therapeutics in GC.

## 2. The FGFR Signaling Pathway and Its Alterations in Gastric Cancer

**Figure 1:**
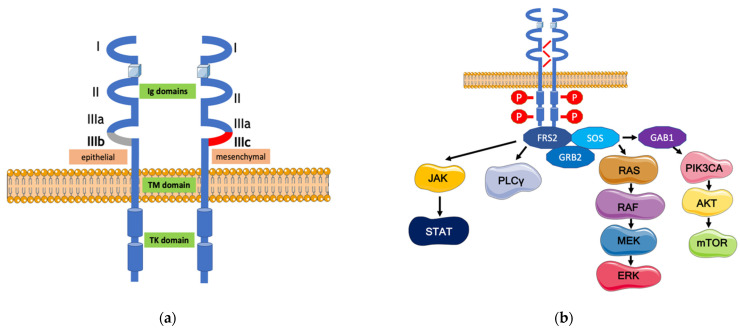
FGFR structure and pathway. (**a**) FGFR structure: FGFR contains three extracellular Ig domains (domains I, II, and III), a single transmembrane helix domain and an intracellular tyrosine kinase domain. The acidic box region between Ig I and II domains can interact with substances other than FGFs. The FGF binding sites are located in domains II and III. For FGFR1-3, the alternative splicing of the second half of the Ig III domain is tissue-dependent. In the case of FGFR4, FGFR contains a single homologous FGFR-IIIc isoform. (**b**) FGFR pathway: Activation of the FGFR tyrosine kinase domain may activate several cellular pathways, including the RAS-RAF-MEK-ERK, the PIK3CA-AKT-mTOR, and the JAK pathways through the FGFR-associated cytosolic docking protein FRS2.

The FGFR has four notable family members, namely FGFR-1, FGFR-2, FGFR-3 and FGFR-4 [20]. The substrate-binding selectivity and tissue distribution of these receptors are different for each receptor [21]. Another source of heterogeneity and ligand-specificity originates from alternative splicing; the four FGFR genes may splice into 48 distinct isoforms [22]. The receptor has three main components: the three extracellular Ig-like domains, a transmembrane helix, and an intracellular tyrosine kinase domain. Alternative splicing of the IgIII loop regulates the FGFR’s ligand specificity, resulting in b- and c-variants of the receptors with different biological effects. These splice variants’ tissue-specific expression controls interactions in embryonic development, tissue maintenance and repair, and cancer. The IIIb variant is principally expressed by epithelial cells, while IIIc is expressed by mesenchymal cells. The formation of the IIIb and IIIc splice variants are mutually exclusive (Figure 1a) [23].

Beyond the heterogeneity of the receptors, several fibroblast growth factors (FGFs) as ligands may activate each receptor, and some FGFs can also activate several other receptors. In particular, FGFs are the most extensive family of growth factor ligands. The structurally related FGF ligands are further subdivided according to their sequence homology [21,24]: FGFs 1–10 and 16–23 are ligands for the FGFR, whereas FGFs 11–14 are cytosolic FGF molecules that operate independently of the receptor [25].

FGFRs may form both homodimers and heterodimers that are stabilized by a heparin sulfate proteoglycan (HSPG) during activation [26]. When FGFRs are activated, their phosphorylated intracellular tyrosine kinase domain may activate several cellular pathways, including the RAS-RAF-MEK-ERK, the PIK3CA-AKT-mTOR and the JAK pathways. The activation of these downstream pathways requires using the FGFR-associated cytosolic docking protein FRS2 and its interaction with other proteins (GRB2, SOS, GAB1, and phospholipase C gamma) [27,28,29,30,31]. Activating these entire signaling pathways may influence angiogenesis, mitogenesis, differentiation, proliferation, changes in tissue homeostasis, and invasion processes (Figure 1b).

Gene fusions, translocations, mutations and amplifications of the FGFR gene have all been reported in cancer. Notably, amplification of the FGF genes was also reported. According to the comprehensive review by Helsten et al., FGFR1 mutations, FGFR2 amplifications and FGFR3 rearrangements are the most common FGFR alterations in GC. These alterations may sometimes be discovered as co-occurring concurrently [32].

A recent article from China summarized the alterations of the FGFR1-4 genes in 5557 solid tumors, including 254 cases of GC. In this analysis, FGFR1-4 aberrations occurred in 12.2% of the GC samples. Amplifications were most prevalent, followed by rearrangements and mutations. Most frequent alterations were detected in the FGFR2 gene, followed by the FGFR1, and to a lesser extent in FGFR3 and FGFR4 genes [33].

Another retrospective analysis identified FGFR alterations in 7% (745/10,582) of GC cases [34]. FGFR2 amplifications are more common in microsatellite-stable (MSS) and TP53 mutant, or MSS/epithelial-mesenchymal transition (EMT) subtypes, according to the Asian Cancer Research Group (ACRG) classification [35]. As per TCGA classification, FGFR2 amplifications are more common in the chromosomal instability (CIN) and genomically stable (GS) subtypes [12,36].

In a study of Chinese patients with GC, FGFR2 amplification was found in 4.1 percent (11/267) of GC cases, especially in the diffuse histology subtype [37]. Another study identified 20% (5/25) of GC to carry the potentially targetable FGFR3-TACC3 (F3T3) fusion [38,39].

Acquired fusions may lead to acquired resistance to FGF-directed therapy. In the case of FGFR2 inhibition of amplified GC, the development of specific fusion proteins may lead to acquired resistance to FGFR2 inhibition. An example of this is the FGFR2-ACSL5 fusion protein, which has been demonstrated to lead to resistance by Kim et al. [40].

According to a preclinical study, another mechanism of acquired resistance to FGFR2 inhibition may be the emergence of a JHDN1D-BRAF fusion, leading to the stimulation of the RAF-MEK pathway. This finding could give a therapeutic basis for employing MEK or RAF inhibitors to prevent resistance from developing or to treat patients who have developed acquired resistance due to the JHDN1D-BRAF fusion [41].

## 3. A Pharmacological Overview on the Anti-FGFR Agents

FGF is a growth protein secreted from fibroblasts and stored near the basal membrane of endothelial cells. As already mentioned, the activation of the FGFR pathway can affect endothelial cell proliferation and differentiation [39], which both are important for embryonic development, wound healing and intra-tumoral angiogenesis [42]. Modulation of endothelial biology and angiogenesis in cancer is common practice since the appearance of vascular endothelial growth factor blockers (VEGFs). Anti-VEGFs are the main anti-angiogenic agents used in the management of gastrointestinal malignancies, even if the results in GC patients were under the expectations [19]. In this regard, the interest in tumor microenvironment as an active player in the process of tumorigenesis and metastatization has led to identify some new molecules that could be targeted with novel drugs. In particular, available data demonstrated that FGF-2/FGFR-2 interaction might bypass the role of the VEGF/VEGFR pathway, acting as a playmaker in the process of angiogenesis and proliferation, especially in GC [19].

Based on this background, several FGFR inhibitors are being developed. TKIs are the most common FGFR antagonists, especially for pretreated cancers with intrinsic resistance to chemotherapy and target therapy [43]. First-generation TKIs are multi-target inhibitors that include the four main isoforms of FGFR and other signaling proteins of the tumor microenvironment, such as VEGFR, KIT, and RET. The multikinase inhibition was associated with severe adverse effects in the landmark trials that limited their clinical use [20]. Since then, refinement of molecular techniques for the design of target-specific molecules and careful selection of patients have demonstrated the potential benefit of FGFR blockade in the growing portfolio of cancer drugs, especially in tumors with poor survival, such as cholangiocarcinoma [44]. A recent study showed that among patients with tumors driven by FGFR aberrations, 76% would be considered ineligible for target therapy due to currently approved indications, comprising 15 different tumor types, potentially susceptible to therapy with TKIs [45].

Among the newer molecules, erdafitinib and pemigatinib are the two TKIs with accelerated regulatory approval, decreasing cell viability by inhibiting FGFR phosphorylation and block signaling. Both medications are contingent upon FGFR alterations; therefore, they are not active in the absence of these alterations [46]. Erdafitinib is an orally active small potent TKI of FGFR1–4. In vivo data shows that it is a potent and selective pan-FGFR inhibitor, including downstream signaling, resulting in a potent anti-proliferative activity, and its intracellular lysosomal localization results in sustained pathway inhibition, with Growth Inhibition 50% (GI50 or IC50) values of 1.2, 2.5, 3.0, and 5.7 nM/L for FGFR1-4 respectively [47]. The results observed in advanced urothelial carcinoma with FGFR2 and FGFR3 genetic alterations granted it accelerated approval by regulating agencies [48].

Pemigatinib is another orally active agent that targets FGFR1, 2 and 3 with IC50 values of less than 2 nM. Pemigatinib also inhibits FGFR4 in vitro at a much higher concentration than those that inhibit FGFR1, 2, and 3. [49]. After the results of the Fight-202 trial, pemigatinib received accelerated approval by the American food and drug administration (FDA) and conditional marketing authorization by the European medicines agency (EMA) for cholangiocarcinoma harboring FGFR2 rearrangements or fusions [50].

Monoclonal antibodies have also represented a breakthrough in advanced esophagogastric and GC protocol designs. Bemarituzumab, an IgG1 antibody targeting FGFR2b ligand-binding domain, blocks ligand-dependent activation of FGFR2b by interfering with the union to FGF; it also mediates antibody-dependent cytotoxicity [51].

## 4. FGFR-Targeted Therapies in Gastric Cancer

### 4.1. Preclinical Studies and Early Phase Clinical Trials

Multikinase inhibitors, pan-FGFR inhibitors, FGFR1-3 inhibitors, selective FGFR inhibitors and antibody-drug conjugates are the most important anti-FGFR agents tested in GC. Here we describe the main results in this setting, according to the drug’s mechanism of action.

#### 4.1.1. Multikinase Inhibitors

Anlotinib, dovitinib, lenvatinib, nintedanib, pazopanib, and ponatinib are the main multikinase inhibitors tested in preclinical studies for GC, whereas no data are available for lucitanib (E-3810) nor masitinib in the preclinical settings in GC.

Anlotinib (VEGFR2-3, FGFR1-4), administered alone or combined with chemotherapy, has a potent inhibitory effect in human xenograft models of multiple cancer types [52]. Anlotinib coupled with an anti-PD-1 antibody was studied in a single-center prospective case-cohort study that enrolled 26 patients with advanced malignancies, including two GC. The combination was safe, and the overall response rate (ORR) was 23.1%. Unfortunately, FGFR status was not tested in this study [53].

Dovitinib (inhibiting VEGFR1/2/3, PDGFRβ, Kit, RET, and FGFR1-3) showed a significant growth inhibitory effect in gastric cells with FGFR2 amplification with IC50 dosages in the submicromolar range [54].

Lenvatinib, which inhibits VEGFR1-3, RET, Kit, and FGFR1-3, demonstrated preclinical efficacy in various human tumor xenograft models [55]. There are case reports available on the combination of lenvatinib with PD-1 inhibitors in GC [56,57].

Nintedanib (inhibiting VEGFR1-3, PDGFRα, and β and FGFR1-3) showed activity in an FGFR2 amplified gastric cell line (KATO-III) [58].

The efficacy of pazopanib (inhibiting VEGFR1-3, c-kit, PDGFR, and FGFRs) has been investigated in 38 GC cell lines. In particular, GC cell lines demonstrating FGFR2 amplification (KATO-III, OCUM-2M, SNU-16, and HSC-39) resulted in significant reductions in cell survival, with IC50 values ranging from 0.1 to 2.0 mol/L. Pazopanib showed no inhibitory effects in cell lines without FGFR amplification. Investigation of the cell cycle showed that FGFR2-amplified cells treated with pazopanib had cell-cycle arrest at the G1-S phase [59]. One patient with FGFR3-amplified mGC responded well to FGFR3 targeting with pazopanib in combination with chemotherapy, according to Limaye et al.’s case report [60].

Ponatinib inhibits Bcr-Abl, VEGFRs and FGFRs; it showed preclinical activity in two FGFR2 amplified gastric cell lines (SNU16, KATO III) by blocking phosphorylation of FGFR2 and the FGFR-associated cytosolic docking protein FRS2 with IC50 values of 20 nmol/L [61].

#### 4.1.2. Pan-FGFR Inhibitors

Futibatinib, LY2874455 and PRN1371 are the main pan-FGFR inhibitors tested in GC; no relevant early preclinical published data regarding erdafitinib (JNJ-42756493) or rogaratinib (BAY 1163877) are available in GC.

In a phase I trial, futibatinib (TAS-120) was tested in 197 patients with advanced solid malignancies. It showed a 13.7 % ORR in the entire cohort, including individuals with secondary resistance to a prior FGFR inhibitor. In the GC cohort, the ORR was 22.2% (95% confidence interval (CI): 2.8–60.0%) [62].

Inhibiting FGFR1-3 by infigratinib in FGFR4 knock-out cell lines demonstrated synergistic activity with 5-fluorouracil. The trial showed that the silencing of FGFR4 might affect the growth characteristics of GC cells (reduced invasiveness, increased apoptosis, decreased ability to proliferate), which may serve as a potential future target [63].

In several tumor xenograft models, including gastric cell lines (SNU-16 and KATO-III), LY-2874455 showed broad-spectrum antitumor activity [64]. Part B of the phase I trial (NCT01212107) of LY-2874455 enrolled 29 patients with GC. FGFR1 and FGFR2 test by fluorescence in situ hybridization (FISH) results were reported in 25 patients. FGFR-amplification has been detected in two patients; both experienced stable disease (SD). Among the 23 FISH-negative patients, one patient had a partial response (PR: 3%), six reached SD (26%), and 16 had progressive disease (PD: 69%) as the best response. No further development of the drug has been reported [65].

Ran et al. recently reported on a promising newly developed FGFR inhibitor: pyrido[1,2-a]pyrimidinone derivative, candidate 23d. At low nanomolar concentrations, the novel drug inhibited phosphorylation of FGFR signaling pathways and triggered cell cycle arrest and apoptosis in a GC xenograft model [66].

#### 4.1.3. FGFR1-3 Inhibitors

AZD4547, CPL304110, Debio1347, infigratinib, and pemigatinib are the main FGFR1-3 inhibitors tested in GC.

The phase Ib trial of AZD4575 (NCT00979134) enrolled 95 participants, but responses were only reported in 15 patients with squamous non-small cell lung cancer [67].

In phase I clinical trial, the oral CPL304110 was tested in patients with advanced solid tumors, including GC. Acquired resistance to the new agent may be reversible by co-targeting MET and Pyk2 [68].

The first-in-human phase I study of the oral debio1347 (NCT1948297) enrolled patients with gastrointestinal cancers and FGFR fusion, including two patients with GC and FGFR2 and FGFR3 fusion. Results of the trial are awaited [69].

Infigratinib (BGJ398) showed preclinical activity in several GC cell lines. The reported efficacy of infigratinib was primarily determined by the expression of FGFR1 as well as FGFR2IIIc [70]. Grygielewicz et al.’s cell-line experiment studied the mechanisms of acquired resistance to FGFR inhibition. The authors modified FGFR2-amplified gastric SNU-16 cell lines to demonstrate EMT (loss of expression of FGFR2 and upregulation of the TGF-β) and resistance to several FGFR inhibitors: the FGFR1-3 inhibitor AZD4547, the pan-FGFR inhibitor infigratinib (BGJ398), and the selective FGFR1 and FGFR3 inhibitor PD173074. Interestingly, the altered SNU-16R cell lines were sensitive to the HER2 inhibitor mubritinib and exhibited epithelial-to-mesenchymal transition-like alterations [71]. In an FGFR-inhibitor-resistant SNU16R-derived xenograft mouse model, knocking out Bcl-2-associated athanogene-3 (BAG3) boosted antitumor effectiveness of infigratinib, suggesting a new therapeutic strategy for GC [72].

#### 4.1.4. Selective FGFR Inhibitors

Alofanib (RPT-835) is the unique selective allosteric FGFR2 inhibitor developed for cancer treatment. After demonstrating preclinical efficacy, it is currently being tested in metastatic GC in an open-label, non-randomized phase Ib trial (NCT04071184) [73].

PD173074is the only FGFR4 selective inhibitor tested in GC. In vitro and in vivo studies of FGFR-nonamplified GC cell lines using the selective FGFR4 inhibitor PD173074 combined with chemotherapy have revealed interesting biological characteristics. In addition to the inhibition of FGFR4, 5-fluorouracil resulted in reduced proliferation. In contrast, when cells were overexpressing FGFR4, the use of FGFR4-inhibition led to an additional inhibition of apoptosis [74].

#### 4.1.5. Antibody–Drug Conjugates

Aprutumab ixadotin (BAY1179470 or BAY1187982) is an intravenous antibody-drug conjugate (ADC) with potential antineoplastic efficacy targeting FGFR2 and coupled to an auristatin-based payload or a thorium-227 isotope (FGFR2-TTC). Unfortunately, after the in vitro and in vivo data of aprutumab ixadotin was released, its first-in-human study reported poor tolerability and terminated early (NCT02368951) [75].

### 4.2. Clinical Trials for FGFR Inhibitors: State of the Art

Based on the preclinical and early phase trials results, FGFR inhibitors have also been tested in later phases of clinical research over the last decades [76].

Historically, dovitinib was one of the first FGFR inhibitors to be investigated in this setting in GC. The phase II GASDOVI-1 trial (NCT01719549) evaluated the safety and efficacy of dovitinib as a later line treatment in mGC patients (≥3 line) with FGFR2 amplification. The trial was completed several years ago; however, to the best of our knowledge, there are no preliminary results reported.

Later, the role of the FGFR1-2-3 inhibitor AZD4547 was also evaluated. The phase II SHINE trial randomized 71 patients to receive paclitaxel (80 mg/m^2^ on days 1, 8, and 15 every 28 days) or AZD4547 (80 mg bid for 14 days every three weeks) as second line treatment for FGFR polysomy of amplified mGC. The trial design has included a 3:2 randomization in the case of FGFR amplification and 1:1 randomization in the case of polysomy. However, the trial failed to show any improvement in the outcome for those patients (PFS: 1.8 vs. 3.5 months in the experimental and control arm, respectively), probably due to the high intratumor heterogeneity with different FGFR expression [77].

Then, the safety and efficacy of the oral multikinase inhibitor anlotinib were tested in the Chinese phase II/III ALTER0503 trial (NCT02461407). The trial randomized mGC patients who have failed the second or higher line of chemotherapy to receive anlotinib or placebo. However, also in this case, the preliminary results are not available at the time of writing. The efficacy and toxicity profile of anlotinib in combination with chemotherapy (S-1, oxaliplatin and capecitabine: SOX schedule) is currently being tested in a phase IV trial in China (ChiCTR1900026291) [78].

Recently, positive results have been reported by using Bemarituzumab in GC. The phase II FIGHT trial investigated the activity of bemarituzumab in the first line treatment for metastatic HER-2 negative gastroesophageal and GC patients [79]. The trial was double-blind, multicentric and randomized; it evaluated 910 mGC patients; of them, 275 patients (30%) had FGFR2b hyperexpressing tumors or FGFR2 gene amplification and were included in the analysis. Among those 275 patients, 155 (149 patients with FGFR2b hyperexpression by immunohistochemistry and 26 patients by circulating tumor DNA (ctDNA)) were randomized to receive chemotherapy (FOLFOX6) ± bemarituzumab (15 mg/Kg every two weeks and 7.5 mg/Kg on day 8). The trial met its endpoints, showing a 2-month improvement in PFS and not reached (NR) OS in the experimental arm (PFS: 9.5 versus 7.4 months in the experimental and control arm, respectively, hazard radio (HR): 0.68, *p* = 0.07; OS: NR versus 12.9 months in the FOLFOX6 arm, HR: 0.58, *p* = 0.03). The responses were also improved (53% in the experimental arm versus 40% in the control arm) as well as the duration of response (12.2 vs. 7.1 months), especially in patients with higher FGFR2b expression. However, the experimental arm showed higher toxicity rates, especially ocular, compared with the chemotherapy alone.

After 12.5 months of follow-up, the long-term results were recently presented to the American Society of Clinical Oncology (ASCO) 2021 meeting, showing a 6-month improvement in OS for patients treated with bemarituzumab (19.2 vs. 13.5 months, HR: 0.6). The benefit was seen especially in patients with FGFR hyper expressing tumor (OS 25.4 months in patients with FGFR2b > 10% of tumor cells versus 11.1 months in the others, HR: 0.41) [80]. Based on these results, the phase III trial is currently ongoing (NCT03343301).

Regarding HER-2 positive tumors, the single-arm, open-label phase II Fighter trial is currently evaluating the safety and activity of pemigatinib in HER-2 metastatic GC patients who progressed after a first-line treatment, including trastuzumab (EudraCT Number 2017-004522-14). The trial was based on the hypothesis that the overexpression of FGFR3 could be linked to the acquired resistance to trastuzumab.

Several trials are currently running to evaluate the role of FGFR inhibitors in GC; Table 1 summarizes the ongoing phase II and III trials in this setting.

## 5. Future Perspectives

FGFR tumorigenic alterations and acquired resistance mutations are recurrent in GC. The current perspectives of new drug development addressing FGFR are exploring either strategies to overcome resistance, or co-target alternative pathways, to restore the sensitivity of FGFR-targeting agents previously received. Table 2 provides an overview of the potential pharmacological targets associated with resistance to anti-FGFR agents.

The challenges in the drug development of new FGFR inhibitors is to identify compounds that are adequately selective, and capable to provide a durable effect. Earlier FGFR inhibitors showed to be poorly specific and quite promiscuous. Limited receptor promiscuity can ultimately improve the safety profile, optimizing the therapeutic efficacy. For instance, this was the case for ponatinib, which is a multi-TKI inhibitor with a spectrum of side effects leading to discontinuation in 10–15% of patients, and significant occurrence of severe adverse events in up to 40% of the patients.

The drug discovery of new anti-FGFR is also focused at preventing the selection pressure of clones generating early therapeutic resistance. New inhibitors can exert activity against multiple acquired and secondary mutations, therefore preventing and overcoming the spectrum of resistance. A classic approach to overcoming resistance to TKI is based on identifying of the pattern of acquired resistance underlying the selection of the primary and secondary unresponsive clones, and the design of molecules to overcome the secondary FGFR mutations. This strategy has been successfully implemented in some disease areas, such as lung cancer and cholangiocarcinoma, but so far has not been pursued yet in GC.

In the aim to develop therapeutic sequences of TKI after TKI with more precise treatments, numerous anti-FGFR compounds are under development (Table 1). Some attractive agents under development are the covalent inhibitors of FGFR, binding the targets outside the catalytic site, serving as allosteric modulators, thus overcoming common gatekeeper mutations.

An emerging strategy to tackle resistant clones is to identify competitive, hyperactive signaling that are elicited by FGFR inhibition as an acquired mechanism of cancer cell adaptation. A well-characterized mechanism in GC is the activation of MAPK signaling, when FGFR is inhibited [81,82]. This means that co-targeting cross-talking pathways may potentiate FGFR inhibition, and improve the therapeutic benefit, as reported with the MEK inhibitor trametinib, in vitro [68].

An alternative mechanism of TKI resistance described for anti-FGFRs are mediated by the sequestration into lysosomes [83]. TKI can diffuse through the lysosomal membranes into the organelles, and sequestered via cation trapping, due to protonation [84]. This creates a gradient toward lysosomes, depauperating the drug off the target(s). Targeting lysosomal storage and biogenesis can result in improved pharmacological activity of FGFR inhibitors. The pharmacological manipulation of the lysosome can be per se a challenge, because they are present in all human cells and govern critical processes of cell physiology. Preventing the acidification of lysosome is a possible target, through membrane disrupting agents. For example, this can result with the use of lysosome acidic pump inhibitors, to reduce the cation trapping [85]. This activity seems to be also exerted by the antimalarial drug hydroxychloroquine. Of interest, key molecules upregulated in cancer cells may play essential roles in lysosomal biogenesis, with the opportunity to better tailor the lysosome-mediated resistance to TKIs. The biogenesis of the lysosome is regulated by the transcription factor EB (TFEB) [86,87]. TFEB, for example, is regulated by the kinase mTORC1 and the phosphatase calcineurin. In GC, the resistance to FGFR inhibitors seems to be mediated by the activation of the mTOR pathway, and the key components of this signaling: the protein TAK1 and mTORC1. The use of mTOR pathway inhibitors and novel anti-TAK1 have been associated with a restored sensitivity to FGFR blockers through a lysosome-disrupting mechanism [88]. Targeting lysosomes biogenesis can be a valuable opportunity to tackle resistance of the GC cells, including through the optimization of FGFR pathway regulators.

Understanding the dynamics of FGFR alterations and the mechanisms of acquired resistance will portray the scenario of a precision medicine approaches, for patients with molecularly-selected GC. Given the recent promising results with some specific inhibitors, evidence on resistance is anticipated to inform the trajectories of drug development and possibly change the therapeutic paradigms in the next years.

Lastly, as the clinical effects of anti-FGFR agents seem very limited according to the current clinical results, further evolution including their combination with checkpoint inhibitors is worth of investigation. Preclinical data gained from breast and lung cancer models showed that FGFR signaling directly influences the immunogenicity of the tumor microenvironment through the migration/expansion/homing of T-lymphocytes. Inhibiting FGFR1 or FGFR4 may contribute to improved response rates of anti-PD-L1 immunotherapies [89,90].

## 6. Conclusions

Despite the advances in cancer treatment, the survival in mGC remains relatively low, partly related to the huge heterogeneity of GC. Therefore, the research regarding a personalized treatment is of fundamental importance in this field in order to improve the outcomes for these patients. Among different targeted agents, the FGFR pathway is one of the novel and promising therapeutic targets proposed to improve the treatment outcomes in GC [19].

FGFR1 mutations, FGFR2 amplifications and FGFR3 rearrangements are the most common FGFR alterations in GC. Several types of FGFR targeting agents were tested or being developed in GC, including multikinase inhibitors, pan-FGFR inhibitors, FGFR1-3 inhibitors, selective FGFR inhibitors and ADC. While most of the studies of multikinase inhibitors were preclinical or single case reports, the phase II GASDOVI-1 trial (NCT01719549) evaluated the safety and efficacy of dovitinib as later line treatment in metastatic GC patients (≥3 line) with FGFR2 amplification, but no results were available despite the end of the trial several years ago. However, the results regarding the use of bemarituzumab in mGC are the most exciting in this field [79,80] and the findings from the ongoing phase III trial are awaited.

Finally, FGFR tumorigenic alterations and acquired resistance mutations are recurrent in GC. The current perspectives of new drug development in this field are exploring the strategies to overcome resistance or co-target alternative pathways, to restore the sensitivity of FGFR-targeting agents previously received.

## Figures and Tables

**Table 1:** Ongoing phase II and III trials testing FGFR inhibitors in gastric cancer.

Name of Drug, Intervention	Tumor Type, Setting, Patient Inclusion/Stratification by Molecular Status	Phase	Study Name, Trial ID	Primary Endpoint
Multikinase Inhibitors				
Anlotinib	Advanced stomach cancer patients, subsequent line of treatment, no molecular stratification	II/III	ALTER0503, NCT02461407	OS
Anlotinib	Advanced stomach cancer patients, subsequent line of treatment, no molecular stratification	II	THALIA, NCT05029102	PFS
TQB2450 /Anlotinib hydrochloride/ Oxaliplatin/Capecitabine	HER2/Neu negative, advanced or metastatic gastric, second-line treatment	N/A	NCT04891900	ORR
TISLELIZUMAB, Anlotinib plus XELOX	Metastatic gastric cancer, first-line, no molecular stratification	III	NCT04963088	MTD, ORR
Tislelizumab + anlotinib	Metastatic gastric cancer, first-line, no molecular stratification	II	NCT04777162	ORR
Anlotinib Plus Toripalimab	Metastatic gastric cancer, subsequent line, no molecular stratification	II	NCT04278222	ORR
Anlotinib	High grade (3), second-line	II	NCT03457844	PFS
Anlotinib Hydrochloride with Nivolumab	Metastatic gastric cancer, second-line, no molecular stratification	II	NCT04503967	ORR
Dovitinib	Advanced gastric cancer, FGFR2 amplification required, subsequent line	II	NCT01719549, GASDOVI-1	RR
Dovitinib Plus Docetaxel	Advanced gastric cancer, first-line, no molecular stratification	I/II	NCT01921673	MTD, PFS
TKI258	Advanced gastric cancer, subsequent line, no molecular stratification	II	NCT01576380	DCR
Lenvatinib + Pembrolizumab	Non-metastatic, fist-line, no molecular classification	II	NCT04745988	MPR rate
Lenvatinib + Pembrolizumab	Advanced gastric cancer, subsequent line, no molecular stratification	II	NCT03321630	ORR, OS
Lenvatinib + Pembrolizumab	Female, advanced ovarian and gastric neoplasms, subsequent line	II	NCT04519151	PFS
Lenvatinib + Pembrolizumab	PD-L1 positive, subsequent line	II	NCT03797326	ORR
Lenvatinib	Advanced gastric cancer, subsequent to prior imatinib or sunitinib	II	NCT04193553	PFS
Pazopanib Hydrochloride	Low- or intermediate-grade neuroendocrine carcinoma, advanced, second-line	II	NCT01841736	PFS
Pan FGFR inhibitors				
Erdafitinib	Advanced gastric cancer, Mandatory FGFR testing, beyond first-line	II	NCT02699606	ORR
Erdafitinib	Advanced Solid tumor, second-line or beyond, FGFR testing	II	NCT02465060	ORR
Futibatinib	Advanced gastric cancer, FGFR testing, beyond first-line	II	NCT04189445	ORR
FGFR1-3 inhibitors				
AZD 4547	Advanced, second line or third line, Mandatory confirmation of FGFR gene amplification	II	NCT01795768	Molecular change as correlated with tumor size change
AZD 4547	Advanced solid tumor, second-line or beyond, based on molecular stratification		NCT02465060, MATCH	ORR
Debio1347	Advanced solid tumor, second-line or beyond, based on molecular stratification	II	NCT03834220	ORR
Infigratinib	Advanced gastric cancer, second-line or beyond, based on FGFR status	II	NCT05019794	ORR
Derazantinib + paclitaxel-ramucirumab/ atezolizumab	Advanced gastric cancer, second-line or beyond, based on FGFR status	I/II	NCT04604132, FIDES-03	ORR
Selective FGFR2-inhibitor				
mFOLFOX6 ± bemarituzumab	Advanced gastric cancer, first-line, based on FGFR status	III	NCT05052801, FORTITUDE-101	OS
Pan FGFR inhibitors				
Erdafitinib	Advanced gastric cancer, Mandatory FGFR testing, beyond first-line	II	NCT02699606	ORR
Erdafitinib	Advanced Solid tumor, second line or beyond, FGFR testing	II	NCT02465060	ORR
Futibatinib	Advanced gastric cancer, FGFR testing, beyond first-line	II	NCT04189445	ORR
FGFR1-3 inhibitors				
AZD 4547	Advanced, second-line or third-line, Mandatory confirmation of FGFR gene amplification	II	NCT01795768	Molecular change as correlated with tumor size change
AZD 4547	Advanced solid tumor, second-line or beyond, based on molecular stratification		NCT02465060, MATCH	ORR
Debio1347	Advanced solid tumor, second-line or beyond, based on molecular stratification	II	NCT03834220	ORR
Infigratinib	Advanced gastric cancer, second-line or beyond, based on FGFR status	II	NCT05019794	ORR
Derazantinib + paclitaxel-ramucirumab/ atezolizumab	Advanced gastric cancer, second-line or beyond, based on FGFR status	I/II	NCT04604132, FIDES-03	ORR

Abbreviations: OS: overall survival; PFS: progression free survival; N/A: not applicable; ORR: overall response rate; Xelox: capecitabine plus oxaliplatin; FGFR: fibroblast growth factor receptor; RR: response rate; DCR: disease control rate; MPR: Major pathological response; MTD: maximum tolerated dose.

**Table 2:** Potential pharmacological targets associated with resistance to anti-FGFR agents.

Potential Target	Mechanism of Resistance
SHP2	Downstream mediator of the FGFR-response
MET	Overexpression is associated with anti-FGFR resistance
Pyk2	Overexpression is associated with anti-FGFR resistance
MET	Secondary activation associated with resistance to anti-FGFR
YAP1	Downstream modulator of the FGFR2 signalling
TAK1	Regulator of the lysosome biogenesis and mediated resistance to anti-FGFR agents
mTORC1	Regulator of the lysosome biogenesis and mediated resistance to anti-FGFR agents
FGF18	Oncogenic stimulation of cancer cells

## Data Availability

Not applicable.
